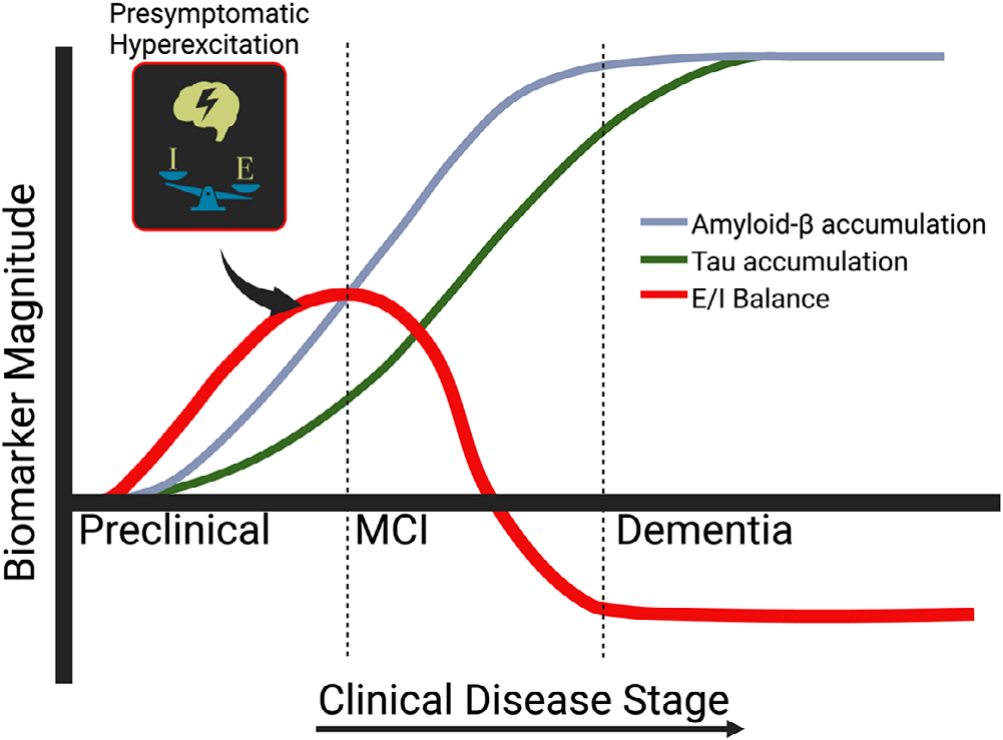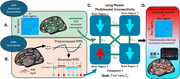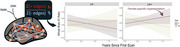# Longitudinal Sex Differences in Excitation–Inhibition Balance in Alzheimer's Disease Risk: A Multimodal Neuroimaging Study

**DOI:** 10.1002/alz70856_107724

**Published:** 2026-01-09

**Authors:** Andrew P Burns, Igor Fortel, Liang Zhan, Orly Lazarov, Scott R. Mackin, Alexander P Demos, Barbara B. Bendlin, Alex Leow

**Affiliations:** ^1^ University of Illinois at Chicago, Chicago, IL, USA; ^2^ University of Chicago, Chicago, IL, USA; ^3^ University of Pittsburgh, Pittsburgh, PA, USA; ^4^ University of Illinois Chicago, Chicago, IL, USA; ^5^ San Francisco Veterans Affairs Medical Center, San Francisco, CA, USA; ^6^ Wisconsin Alzheimer's Disease Research Center, School of Medicine and Public Health, University of Wisconsin, Madison, WI, USA

## Abstract

**Background:**

Excessive neural hyperexcitation has been implicated in early cognitive decline and progression to Alzheimer's disease (AD). Restoring the balance between excitation and inhibition (E/I) with interventions like levetiracetam may offer clinical benefits, particularly for those at heightened risk. Recent cross‐sectional studies suggest that female APOE‐ε4 carriers may be especially vulnerable to hyperexcitation, but longitudinal evidence remains limited. We therefore investigated whether E/I dysregulation over time differs by sex and APOE‐ε4 status in older adults who were cognitively unimpaired at baseline. Figure 1 illustrates the concept of early hyperexcitation preceding AD symptoms.

**Method:**

We analyzed multimodal MRI data (resting‐state functional MRI and diffusion‐weighted imaging) from 106 older adults with at least one cognitively unimpaired scan and three or more longitudinal sessions. Most sessions were rated as clinically unimpaired (CDR = 0), though a subset transitioned to early mild cognitive impairment (CDR = 0.5 or 1). We applied an inverse Ising model regularized by empirical structural connectivity (Figure 2) to derive a whole‐brain excitation‐inhibition ratio (EIR). Linear mixed modeling tested whether EIR trajectory varied by sex, binary APOE‐ε4 status, age at first scan, and time since first scan.

**Result:**

A significant three‐way interaction (Figure 3) indicated that female APOE‐ε4 carriers demonstrate an elevated hyperexcitable EIR trajectory (*p* = 0.018). Pairwise comparisons further showed higher EIR slopes for female ε4 carriers compared to female non‐carriers (*p* = 0.042). This effect remained significant after adjusting for age, time, and amyloid status, though it was somewhat diminished in participants with fewer longitudinal observations. Regional analyses focusing on default mode and limbic networks found higher baseline excitatory tone in females (*p* = 0.02), aligning with the global results.

**Conclusion:**

These findings provide longitudinal support for a heightened susceptibility to hyperexcitation in female APOE‐ε4 carriers, underscoring the importance of sex and genetic risk in preventive and therapeutic strategies for AD. Our multimodal approach integrating structural and functional network data highlights E/I balance as a promising biomarker and treatment target, with levetiracetam representing one potential intervention. Larger studies are needed to confirm how sex‐ and genotype‐specific E/I dysregulation influences dementia risk and therapeutic efficacy.